# Fractal analysis of left ventricular trabeculae in post-STEMI: from acute to chronic phase

**DOI:** 10.1186/s13244-024-01641-8

**Published:** 2024-03-18

**Authors:** Ruo-Yang Shi, Rui Wu, Jinjun Ran, Lang-Lang Tang, Luke Wesemann, Jiani Hu, Liang Du, Wei-Jun Zhang, Jian-Rong Xu, Yan Zhou, Lei Zhao, Jun Pu, Lian-Ming Wu

**Affiliations:** 1 Department of Radiology, School of Medicine, Ren Ji Hospital, Shanghai Jiao Tong University, No. 160, Pujian Road, Shanghai, 200127 China; 2 Jiading Branch, Ren Ji Hospital, Shanghai Jiao Tong University School of Medicine, Shanghai, China; 3 School of Public Health, Shanghai Jiao Tong University School of Medicine, Shanghai, China; 4 Department of Radiology, Longyan First Hospital of Fujian Medical University, Long Yan, Fu Jian, China; 5 Department of Radiology, Wayne State University, Detroit, MI USA; 6 Shanghai Robotics Institute, Shanghai University, Shanghai, China; 7 School of Mechanical Engineering, Shanghai Jiao Tong University, Shanghai, China; 8 Department of Radiology, An Zhen Hospital, Capital Medical University, No. 2 Anzhen Road, Beijing, 100029 China; 9 Department of Cardiology, School of Medicine, Ren Ji Hospital, Shanghai Jiao Tong University, No. 160, Pujian Road, Shanghai, 200127 China

**Keywords:** Magnetic resonance imaging, Cine, ST elevation myocardial infarction, Myocardium, Fractals

## Abstract

**Purpose:**

The temporal evolution of ventricular trabecular complexity and its correlation with major adverse cardiovascular events (MACE) remain indeterminate in patients presenting with acute ST elevation myocardial infarction (STEMI).

**Methods:**

This retrospective analysis enrolled patients undergoing primary percutaneous coronary intervention (pPCI) for acute STEMI, possessing cardiac magnetic resonance (CMR) data in the acute (within 7 days), subacute (1 month after pPCI), and chronic phases (6 months after pPCI) from January 2015 to January 2020 at the three participating sites. Fractal dimensions (FD) were measured for the global, infarct, and remote regions of left ventricular trabeculae during each phase. The potential association of FD with MACE was analyzed using multivariate Cox regression.

**Results:**

Among the 200 analyzed patients (182 men; median age, 61 years; age range, 50–66 years), 37 (18.5%) encountered MACE during a median follow-up of 31.2 months. FD exhibited a gradual decrement (global FD at acute, subacute, and chronic phases: 1.253 ± 0.049, 1.239 ± 0.046, 1.230 ± 0.045, *p* < 0.0001), with a more pronounced decrease observed in patients subsequently experiencing MACE (*p* < 0.001). The global FD at the subacute phase correlated with MACE (hazard ratio 0.89 (0.82, 0.97), *p* = 0.01), and a global FD value below 1.26 was associated with a heightened risk.

**Conclusion:**

In patients post-STEMI, the global FD, serving as an indicator of left ventricular trabeculae complexity, independently demonstrated an association with subsequent major adverse cardiovascular events, beyond factors encompassing left ventricular ejection fraction, indexed left ventricular end-diastolic volume, infarct size, heart rate, NYHA class, and post-pPCI TIMI flow.

**Critical relevance statement:**

In patients who have had an ST-segment elevation myocardial infarction, global fractal dimension, as a measure of left ventricular trabeculae complexity, provided independent association with subsequent major adverse cardiovascular event.

**Key points:**

• Global and regional FD decreased after STEMI, and more so in patients with subsequent MACE.

• Lower global FD at the subacute phase and Δglobal FD from acute to subacute phase were associated with subsequent MACE besides clinical and CMR factors.

• Global FD at the subacute phase independently correlated with MACE and global FD value below 1.26 was associated with higher risk.

**Graphical Abstract:**

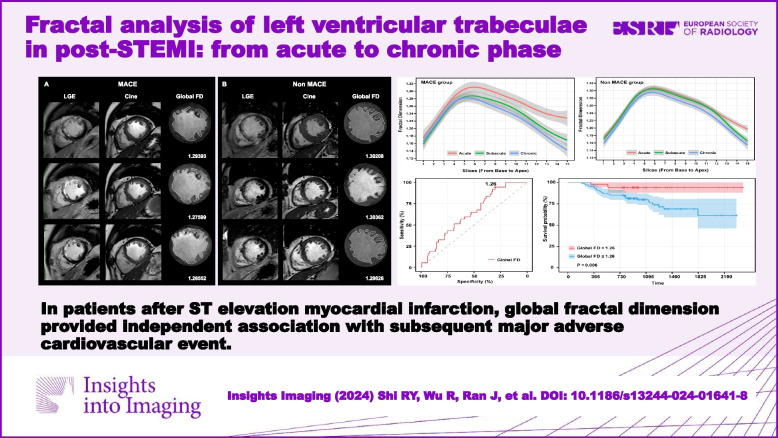

**Supplementary Information:**

The online version contains supplementary material available at 10.1186/s13244-024-01641-8.

## Introduction

Ventricular trabeculation constitutes an intricate network of muscle fibers, exhibiting diverse sizes and attachment modes to the inner myocardial wall. The morphology of trabeculae is presumed to contribute to effective nutrient and oxygen diffusion from the blood pool, as well as an efficient intraventricular flow pattern [1–3]. The functional role of ventricular trabeculation in cardiac performance remains incompletely understood at present.

The complexity of trabeculation in the left and right ventricles of the heart can be quantified using the fractal dimension (FD) through magnetic resonance imaging [4, 5]. Elevated FD stands as an independent predictor of all-cause mortality and composite events in patients with hypertrophic cardiomyopathy [4]. Moreover, it has been correlated with myocardial remodeling in patients with pulmonary hypertension [5] and associated with systolic dysfunction of the left ventricle in patients with left ventricular noncompaction [6].

Despite significant attention directed towards myocardial infarction (MI), ST elevation myocardial infarction (STEMI) continues to be linked with substantial morbidity and mortality on a global scale [7]. To the best of our knowledge, trabecular complexity and its temporal pattern have not been documented in patients with acute myocardial infarction (AMI). Following acute STEMI, the ventricular wall and volume undergo gradual changes as part of compensatory or decompensatory adaptation, but the variation of ventricular trabeculae has not been explored previously. In this context, we undertook a retrospective analysis to scrutinize changes in FD through cardiac magnetic resonance (CMR) from the acute to chronic phases of STEMI subjects. Additionally, we investigated the potential association between FD changes and subsequent major adverse cardiovascular events (MACE) during long-term follow-up.

## Methods and materials

### Study population

This retrospective study encompassed consecutive adult patients who underwent primary percutaneous coronary intervention (pPCI) for acute STEMI within the timeframe spanning January 2015 to June 2020 at three distinct hospitals. To qualify for inclusion in the final analysis, patients were required to have accessible CMR data obtained during the acute phase (within 7 days after pPCI), the subacute phase (1 month after pPCI), and the chronic phase (6 months after pPCI). The diagnosis of acute STEMI was established based on the presence of elevated ST segments of 0.1 mV or more in at least two contiguous leads, coupled with an elevation in cardiac troponin levels. Major adverse cardiovascular events (MACE) were defined as a composite outcome comprising re-infarction, hospitalization for heart failure, stroke, and cardiovascular death. Follow-up data were censored in June 2022. In instances where multiple events occurred in a single patient, only the initial event was considered for endpoint analysis. Patients with a documented history of prior myocardial infarction or concurrent non-ischemic cardiomyopathy were excluded from the analysis, as were those with a history of MACE preceding the second CMR examination.

This retrospective study protocol was approved by the local institutional review board and written informed consent was waived.

### CMR

Images were obtained using 3.0-Tesla MRI scanners (manufactured by Philips, Siemens, and General Electric). The standard analysis comprised steady-state free-precession (SSFP) cine images, T2-weighted imaging with fat suppression (T2WFS) images, and late gadolinium enhancement (LGE) images.

The evaluation of left ventricular volume, mass, and function as well as infarct size, edema, microvascular obstruction (MVO), and intramyocardial hemorrhage (IMH), was performed using CVI42 (Circle Cardiovascular Imaging Inc. Calgary, Canada) by two independent radiologists with 4 and 6 years of experience, respectively. The calculation of the transmural extent of the infarct from LGE images was expressed as a percentage, obtained by dividing infarct wall thickness by regional wall thickness. Comprehensive MRI acquisition protocols and image assessments are outlined in the Supplementary material.

### Fractal analysis

**Fig. 1:**
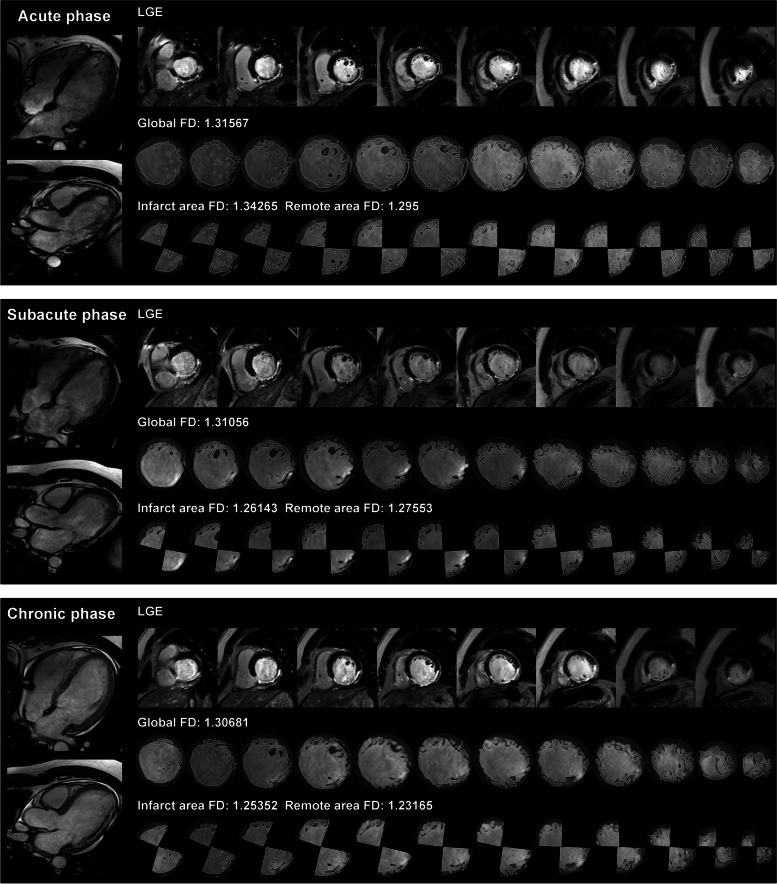
Case example. Cardiac magnetic resonance (CMR) images of a 37-year-old man with acute right coronary artery (RCA) infarction. Primary percutaneous coronary intervention (pPCI) was conducted 24 h after the initial symptoms emerged. CMR was performed on day 3, 1 month, and 6 months. No subsequent MACE occurred within the 25-month follow-up

Fractal analysis was carried out using FracAnalyse [8], a custom-written code employing Matlab software (Mathworks, Natick, Mass) with established robustness, as previously documented [4–6]. FD values were quantified as follows: (1) global FD encompassing the entire left ventricle, and (2) regional FD comprising FD values specific to the infarct and remote areas. All FD measurements were conducted during the end-diastolic phase of short-axis cine images. The global FD value represented the average across all slices from the base to the apex. For each slice, an elliptical or polygonal region of interest (ROI) was manually delineated within the midwall of the left ventricular myocardium. Regional FD values for the infarct and remote areas were manually outlined in partial slices containing both areas. To mitigate the impact of ROI shape on FD values, regional FD ROIs were consistently depicted as 90° sector shapes. The remote FD ROI was defined as a sector 180° from the infarct ROI, displaying no visible evidence of infarction and edema. The regional FD value was defined as the average across all measured slices. Examples of ROI sketches are presented in Fig. 1. The algorithm for FD calculation has been comprehensively described in previous publications [5, 9, 10]. Due to variations in the number of global short-axis slices among subjects, FD values were interpolated to a 15-slice model. The fractal analysis was executed by two readers, possessing 4 and 6 years of CMR diagnosis experience. In addition to FD measures for the global, infarct, and remote areas, the absolute change in FD measures (ΔGlobal FD, ΔInfarct area FD, and ΔRemote area FD) from the acute phase to the subacute and chronic phases was calculated. To assess interobserver reproducibility, global and regional FD measurements for 20 patients at each phase were independently analyzed by two radiologists, with 4 and 6 years of experience, respectively.

### Statistical analysis

Continuous variables with a normal distribution were compared between subjects with and without MACE using Student’s *t* test and are presented as mean ± standard deviation. Continuous variables with a skewed distribution were analyzed using the Mann–Whitney *U* test and are expressed as median and inter-quartile range (IQR). Categorical variables were compared using the *χ*^2^ test or Fisher exact test, as appropriate. FD in every phase was compared using pairwise one-way ANOVA with Tukey post hoc test. The correlation between variables was assessed with either Pearson or Spearman correlation analysis. Interobserver agreement was evaluated by two-way intraclass correlation and Bland–Altman analysis. Risk factors for MACE were assessed using univariable and multivariable Cox regression analysis. In multivariable binary logistic and Cox regression analyses, variables were selected using backward stepwise regression with Akaike information criterion (AIC), and the model with the minimum AIC value was reported (Model 1 in Table 4). The factors included in the initial multivariable regression analyses were based on the results of univariable regression as well as previous studies [11–13], encompassing patient characteristics and CMR parameters. Significant FD measures identified in univariable regression analysis were subsequently added to the model to assess their incremental value. The change in models was assessed by Harrell’s C-statistics. The Integrated Discrimination Index (IDI) and continuous Net Reclassification Improvement (cNRI) were calculated for models with and without FD measures. Receiver operating characteristic (ROC) analysis was employed to evaluate the model performance, and the best cut-off value was selected based on the Youden index. Survival curves were generated using the Kaplan–Meier method and compared using the log-rank test. Statistical significance was defined as *p* < 0.05 (2-sided). All analyses were conducted using R (version 4.0.3) with RStudio (version 1.3.959).

## Results

### Characteristics of the study population

**Fig. 2:**
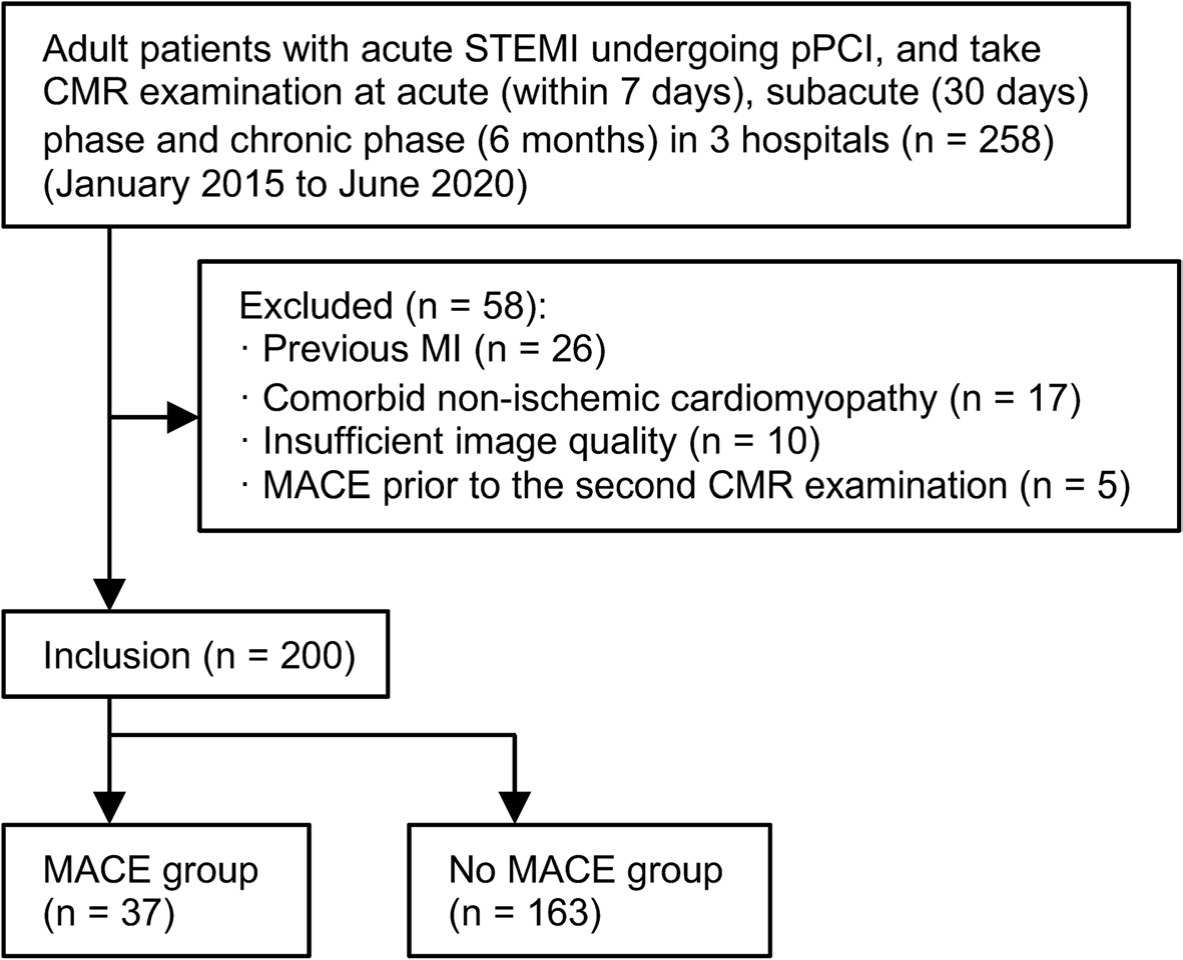
Study inclusion flowchart. STEMI: ST elevation myocardial infarction, *pPCI* primary percutaneous coronary intervention, *CMR* cardiac magnetic resonance, *MI* myocardial infarction, *MACE* major adverse cardiovascular event

The initial screening of the database identified a total of 258 STEMI patients undergoing pPCI with CMR in all three phases. Fifty-eight cases were excluded for the following reasons: previous myocardial infarction (MI) (*n* = 26), comorbid non-ischemic cardiomyopathy (*n* = 17), insufficient image quality for analysis (*n* = 10), and MACE prior to the second CMR examination (*n* = 5). The final analysis included 200 patients across all three phases: the acute phase (2.1 ± 1.5 days), subacute phase (29.6 ± 3.4 days), and chronic phase (≥ 6 months). The flow diagram illustrating inclusion and nonattendance reasons is summarized in Fig. 2.

During the follow-up period (median 31.2 months, IQR 26.9–41.6 months), MACE occurred in 37 (18.5%) patients. Specific events included re-infarction (*n* = 11), hospitalization for heart failure (*n* = 23), and cardiovascular death (*n* = 3). Subjects with subsequent MACE exhibited a higher New York Heart Association (NYHA) functional class and a lower thrombolysis in myocardial infarction (TIMI) flow level after pPCI (Table 1).

**Table 1:** Demographic and baseline characteristics

Characteristics	All subjects (*n* = 200)	Non MACE (*n* = 163)	MACE (*n* = 37)	*p* value
Age (y)	61 (50, 66)	61 (50, 66)	59 (51, 66)	0.94
Male sex, n (%)	182 (91.0)	150 (92.0)	32 (86.5)	0.31
Body mass index (kg/m^2^)	24.7 ± 2.9	24.9 ± 2.8	24.0 ± 3.4	0.12
Body surface area (m^2^)	1.85 ± 0.16	1.86 ± 0.15	1.81 ± 0.2	0.11
Cardiac risk factors, n (%)				
Hypertension	117 (58.5)	93 (57.1)	24 (64.9)	0.47
Dyslipidemia	43 (21.5)	36 (22.1)	7 (18.9)	0.82
Diabetes mellitus	56 (28)	43 (26.4)	13 (35.1)	0.30
Current smoker	123 (61.5)	99 (60.7)	24 (64.9)	0.71
SBP (mmHg)	126.0 ± 18.0	126.1 ± 17.6	125.5 ± 19.7	0.99
DBP (mmHg)	76.5 ± 11.3	76.6 ± 11.6	76.9 ± 10.0	0.98
NYHA functional class, n (%)				**0.02**
I	182 (91)	151 (92.6)	31 (83.8)	
II	16 (8)	12 (7.4)	4 (10.8)	
III	1 (0.5)	0 (0)	1 (2.7)	
IV	1 (0.5)	0 (0)	1 (2.7)	
Pain-to-balloon time (h)	4 (2.5, 8)	4 (2.75, 8)	5 (2, 6)	0.99
Culprit lesion, n (%)				0.49
LAD	152 (76)	122 (74.8)	30 (81.1)	
LCX	13 (6.5)	10 (6.1)	3 (8.1)	
RCA	35 (17.5)	31 (19.1)	4 (10.8)	
TIMI flow at baseline, n (%)				0.22
0	131 (65.5)	104 (63.8)	27 (73.0)	
1	10 (5)	10 (6.1)	0 (0)	
2	14 (7)	10 (6.1)	4 (10.8)	
3	45 (22.5)	39 (23.9)	6 (16.2)	
TIMI flow after pPCI, n (%)				**0.043**
0	1 (0.5)	0 (0)	1 (2.7)	
1	3 (1.5)	2 (1.2)	1 (2.7)	
2	9 (4.5)	5 (3.1)	4 (10.8)	
3	187 (93.5)	156 (95.7)	31 (83.8)	

Data are mean ± standard deviation for normally distributed variables, median and interquartile range (in parentheses) for skewed variables

*MACE* Major adverse cardiovascular events, *SBP* Systolic blood pressure, *DBP* Diastolic blood pressure, *LAD* Left anterior descending, *LCX* Left circumflex artery, *RCA* Right coronary artery, *TIMI* Thrombolysis in myocardial infarction

### CMR *f*indings

From the acute phase to the chronic phase, CMR parameters were compared between the MACE and Non-MACE groups. In the acute phase, heart rate, indexed left ventricular end-diastolic volume (LVEDVi), indexed left ventricular end-systolic volume (LVESVi) and infarct size were significantly higher in the MACE group compared to those in the Non-MACE group (Table 2). In the subacute and chronic phases, left ventricular ejection fraction (LVEF) was lower in the MACE group than in the Non-MACE group. The heart rate of the MACE group in the subacute phase was higher than that of the Non-MACE group. In the chronic phase, no significant difference was found between the heart rates of the two groups. The infarct size of the MACE group remained larger than that of the Non-MACE group from the acute to the chronic phase (Acute phase Non-MACE: 24.51% ± 10.46%, MACE: 35.33% ± 12.97%, *p* < 0.001; Subacute phase Non-MACE: 19.41% ± 8.97%, MACE: 29.09% ± 12.18%, *p* < 0.001; Chronic phase Non-MACE: 17.38% ± 8.65%, MACE: 26.48% ± 12.07%, *p* < 0.001). No significant difference was found between papillary infarction in the two groups.

**Table 2:** Left ventricular characteristics and fractal dimension in each phase

Characteristics	All subjects (*n* = 200)	Non MACE (*n* = 163)	MACE (*n* = 37)	*p* value
Acute phase				
CMR parameters				
Heart rate (beats/min)	74.6 ± 11.3	73.8 ± 11.4	78.2 ± 10.6	**0.03**
LVEF (%)	49.13 ± 11.23	49.8 ± 10.58	46.21 ± 13.49	0.14
LVMi (g/m^2^)	64.61 ± 12.23	63.76 ± 10.92	68.34 ± 16.51	0.12
LVEDVi (mL/m^2^)	81.22 ± 20.44	78.98 ± 16.64	91.12 ± 30.66	**0.03**
LVESVi (mL/m^2^)	42.54 ± 19.98	40.25 ± 14.39	52.63 ± 33.85	**0.04**
LVSVi (mL/m^2^)	38.68 ± 9.36	38.73 ± 9.11	38.48 ± 10.52	0.90
Infarct size (% of LV)	26.51 ± 11.71	24.51 ± 10.46	35.33 ± 12.97	** < 0.001**
Infarct transmural extent (%)	95 (90, 100)	95 (90, 100)	100 (95, 100)	0.12
Edema size (% of LV)	37.02 ± 13.54	36.5 ± 13.57	39.32 ± 13.38	0.25
LV thrombus, n (%)	28, 14	20, 12.3	8, 21.6	0.19
LV aneurysm, n (%)	24, 12	20, 12.3	4, 10.8	0.99
MVO/IMH, n (%)	130, 65	109, 66.9	21, 56.8	0.24
Papillary infarction, n (%)	34, (17)	30 (18.4)	4 (10.8)	0.46
FD measures				
Global FD	1.253 ± 0.049	1.251 ± 0.048	1.260 ± 0.052	0.35
Infarct area FD	1.209 ± 0.08	1.211 ± 0.079	1.199 ± 0.070	0.37
Remote area FD	1.243 ± 0.058	1.244 ± 0.058	1.236 ± 0.054	0.44
Subacute phase				
CMR parameters				
Heart rate (beats/min)	67.8 ± 10.7	66.9 ± 10.6	71.6 ± 10.4	**0.02**
LVEF (%)	50.35 ± 11.54	51.37 ± 10.67	45.86 ± 14.08	**0.03**
LVMi (g/m^2^)	60.65 ± 12.34	59.91 ± 11.19	63.92 ± 16.28	0.16
LVEDVi (mL/m^2^)	82.53 ± 21.85	79.46 ± 16.82	96.08 ± 33.69	**0.006**
LVESVi (mL/m^2^)	42.29 ± 20.28	39.29 ± 14.48	55.53 ± 33.3	**0.006**
LVSVi (mL/m^2^)	40.24 ± 9.09	40.17 ± 9.24	40.55 ± 8.51	0.81
Infarct size (% of LV)	21.2 ± 10.32	19.41 ± 8.97	29.09 ± 12.18	** < 0.001**
Infarct transmural extent (%)	95 (75, 100)	90 (75, 100)	100 (90, 100)	0.10
LV thrombus, n (%)	11, 5.5	10, 6.1	1, 2.7	0.49
LV aneurysm, n (%)	34, 17	26, 15.9	8, 21.6	0.47
MVO/IMH, n (%)	80, 40	62, 38.0	18, 48.6	0.26
FD measures				
Global FD	1.239 ± 0.046	1.244 ± 0.048	1.219 ± 0.033	** < 0.001**
Infarct area FD	1.178 ± 0.069	1.182 ± 0.071	1.16 ± 0.057	**0.01**
Remote area FD	1.239 ± 0.056	1.243 ± 0.056	1.219 ± 0.05	**0.01**
ΔGlobal FD	-0.014 ± 0.037	-0.008 ± 0.034	-0.041 ± 0.037	** < 0.001**
ΔInfarct area FD	-0.031 ± 0.036	-0.029 ± 0.036	-0.033 ± 0.036	0.15
ΔRemote area FD	-0.003 ± 0.034	-0.001 ± 0.033	-0.017 ± 0.035	**0.01**
Chronic phase				
CMR parameters				
Heart rate (beats/min)	66.3 ± 11.0	65.6 ± 10.7	69.2 ± 11.9	0.10
LVEF (%)	50.1 ± 16.01	51.31 ± 16.09	44.8 ± 14.71	**0.02**
LVMi (g/m^2^)	59.62 ± 12.04	58.84 ± 10.4	63.09 ± 17.29	0.16
LVEDVi (mL/m^2^)	82.74 ± 22.52	79.48 ± 17.65	97.11 ± 33.77	**0.004**
LVESVi (mL/m^2^)	42.57 ± 22.11	39.19 ± 16.06	57.5 ± 35.51	**0.002**
LVSVi (mL/m^2^)	40.17 ± 9.87	40.29 ± 10	39.62 ± 9.4	0.70
Infarct size (% of LV)	19.06 ± 9.99	17.38 ± 8.65	26.48 ± 12.07	** < 0.001**
Infarct transmural extent (%)	95 (75, 100)	95 (75, 100)	100 (85, 100)	0.14
LV thrombus, n (%)	6, 3	5, 3.1	1, 2.7	0.99
LV aneurysm, n (%)	35, 17.5	26, 15.9	9, 24.3	0.25
MVO/IMH, n (%)	33, 16.5	27, 16.5	6, 16.2	0.72
FD measures				
Global FD	1.230 ± 0.045	1.233 ± 0.047	1.216 ± 0.034	**0.01**
Infarct area FD	1.169 ± 0.068	1.174 ± 0.069	1.149 ± 0.055	**0.02**
Remote area FD	1.233 ± 0.057	1.237 ± 0.058	1.217 ± 0.048	**0.04**
ΔGlobal FD	-0.023 ± 0.047	-0.018 ± 0.046	-0.044 ± 0.04	**0.001**
ΔInfarct area FD	-0.040 ± 0.044	-0.037 ± 0.044	-0.040 ± 0.031	0.26
ΔRemote area FD	-0.009 ± 0.044	-0.007 ± 0.045	-0.019 ± 0.037	0.09

Data are mean ± standard deviation for normally distributed variables, median and interquartile range (in parentheses) for skewed variables

*MACE* Major adverse cardiovascular events, *LVEF* Left ventricular ejection fraction, *LVEDVi* Left ventricular end-diastolic volume index, *LVESVi* Left ventricular end-systolic volume index, *LVMi* Left ventricular mass index, *LVSVi* Left ventricular stroke volume index, *MVO* Microvascular obstruction, *IMH* Intramyocardial hemorrhage, *LVT* Left ventricular thrombus, *FD* Fractal dimension

### Changes in FD from acute to chronic phase

**Fig. 3:**
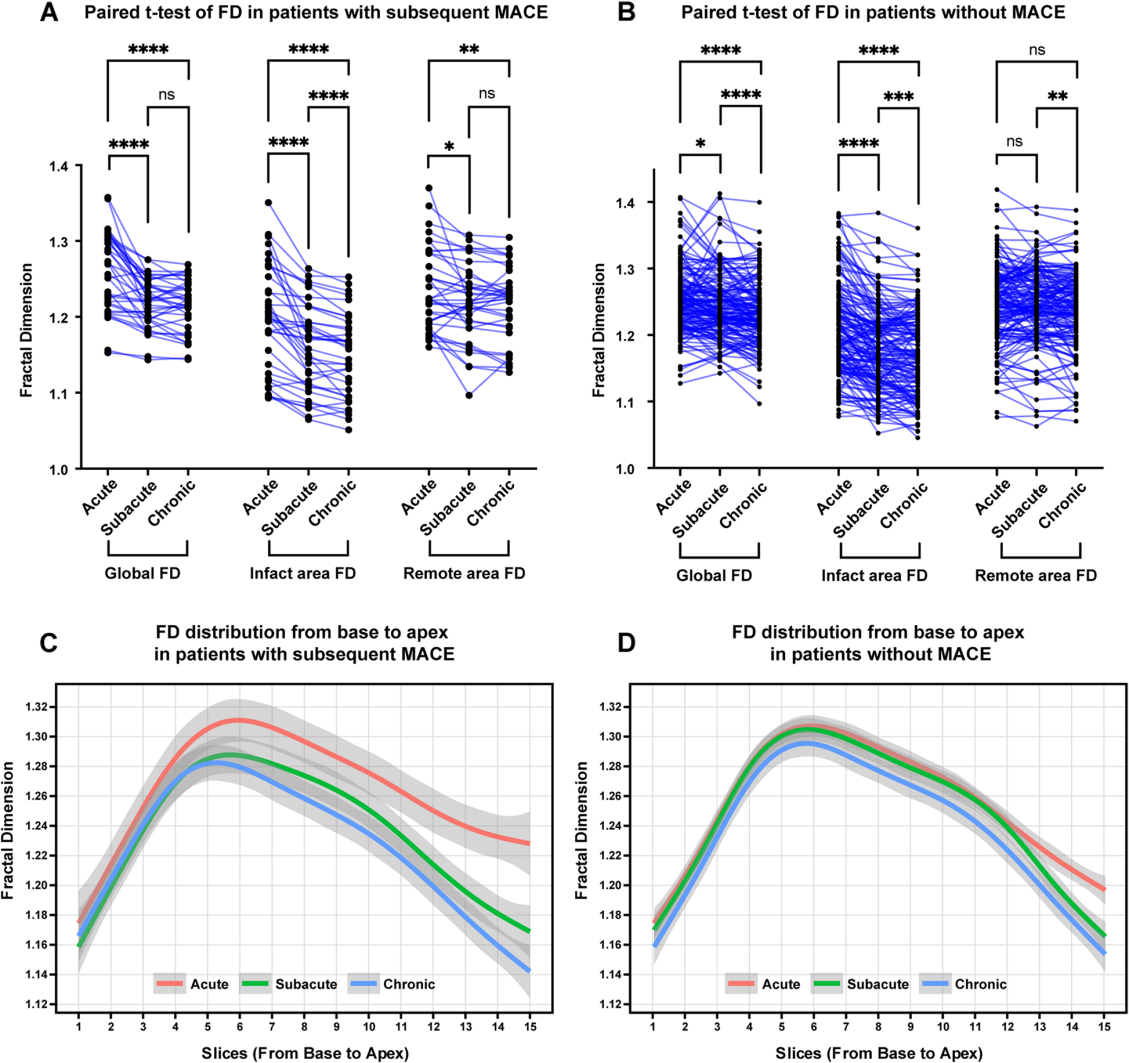
Fractal dimension (FD) in subjects with versus without major adverse cardiovascular events (MACE). **A** Paired *t*-test of FD in patients with subsequent MACE. **B** Paired *t*-test of FD in patients without MACE. **C** FD distribution from base to apex in patients with subsequent MACE. **D** FD distribution from base to apex in patients without MACE. *, *p* < 0.05; **, *p* < 0.01; ***, *p* < 0.001; ****, *p* < 0.0001; ns, not significant

FD values for each phase are summarized in Table 2. In the acute phase, FDs in the MACE and Non-MACE groups were comparable (Global FD Non-MACE 1.251 ± 0.048, MACE 1.260 ± 0.052, *p* = 0.35). However, FD values exhibited continuous changes from the acute to the chronic phase, as illustrated in Fig. 3. Over this period, FD values demonstrated a progressive decrease, with slight variations in this decreasing trend between the MACE and Non-MACE groups. In both groups, global FD significantly decreased, yet there were distinctions in the timing of this decrease. In the MACE group, the decrease was primarily concentrated from the acute phase to the chronic phase, whereas in the Non-MACE group, the decrease was mainly concentrated from the subacute phase to the chronic phase (each phase of MACE group: 1.260 ± 0.052, 1.219 ± 0.033, 1.216 ± 0.034, acute to subacute *p* < 0.0001, subacute to chronic *p* = 0.32; Non-MACE group: 1.251 ± 0.048, 1.244 ± 0.048, 1.233 ± 0.047, acute to subacute *p* = 0.01, subacute to chronic *p* < 0.0001). This trend is also evident in the distribution maps of FD from the acute to chronic phases (Fig. 3C, D). The FD of the infarcted area continuously decreased from the acute to subacute and chronic phases, indicating a continuous atrophy or reduction in the corresponding regional trabecular structure (each phase of MACE group: 1.199 ± 0.070, 1.16 ± 0.057, 1.149 ± 0.055, acute to subacute *p* < 0.0001, subacute to chronic *p* < 0.0001; each phase of Non-MACE group: 1.211 ± 0.079, 1.182 ± 0.071, 1.174 ± 0.069, acute to subacute *p* < 0.0001, subacute to chronic *p* = 0.0006). However, the decreasing trend of remote area FD was notably different. In the MACE group, remote area FD significantly decreased, mainly occurring from the acute to subacute phase, while in the Non-MACE group, although remote area FD slightly decreased from the subacute to the chronic phase, the overall change was not significant (each phase of MACE group: 1.236 ± 0.054, 1.219 ± 0.05, 1.217 ± 0.048, acute to subacute *p* = 0.016, subacute to chronic *p* = 0.72; each phase of Non-MACE group: 1.244 ± 0.058, 1.243 ± 0.056, 1.237 ± 0.058, acute to subacute *p* = 0.98, subacute to chronic *p* = 0.008).

**Fig. 4:**
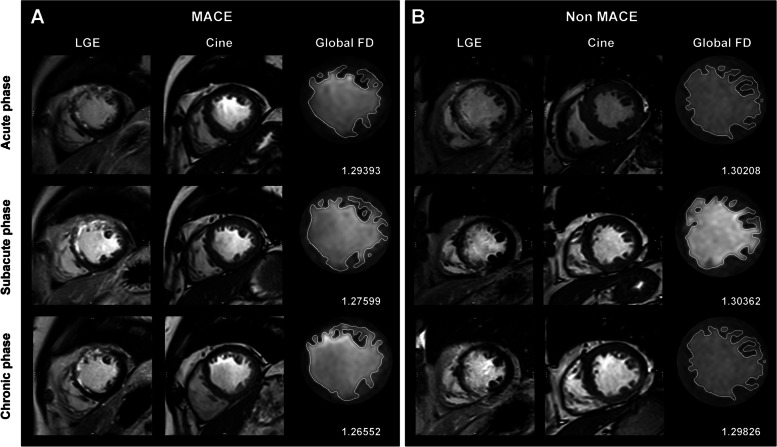
Case examples of patients with subsequent major adverse cardiovascular events (MACE) (**A**) and without MACE (**B**). Only mid-ventricular level images of late gadolinium enhancement, cine, and global FD figures at acute, subacute, and chronic phases are summarized in this figure. **A** A 50-year-old man with acute left anterior artery (LAD) infarction. Global FD decreased from the acute phase to the chronic phase. Heart failure occurred 19 months after primary percutaneous coronary intervention. **B** A 65-year-old man with acute LAD infarction. Global FD slightly elevated in the subacute phase and then decreased in the chronic phase. No subsequent MACE occurred within the 38-month follow-up

This differing decreasing trend resulted in variations in the FD values and change values between the subacute and chronic phases of the two groups, primarily concentrated in the subacute phase. During the subacute phase, statistically significant differences were observed in global, infarct area, and remote area FD between the two groups (Fig. 4). Concerning change values, there was not much difference in the infarct area between the two groups, but significant differences were noted in the global and remote areas (global FD of Non-MACE and MACE: 1.244 ± 0.048, 1.219 ± 0.033, *p* < 0.001; infarct area FD of Non-MACE and MACE: 1.182 ± 0.071, 1.16 ± 0.057, *p* = 0.01; remote area FD of Non-MACE and MACE: 1.243 ± 0.056, 1.219 ± 0.05, *p* = 0.01; ΔGlobal FD of Non-MACE and MACE: -0.008 ± 0.034, -0.041 ± 0.037, *p* < 0.001, ΔRemote area FD of Non-MACE and MACE: -0.001 ± 0.033, -0.017 ± 0.035, *p* = 0.01).

The intraclass correlation coefficients of global, infarct area, and remote area FD were 0.952 (95% confidence interval [CI] 0.897, 0.975), 0.913 (95% CI 0.606, 0.967), and 0.922 (95% CI 0.873, 0.953), respectively. Results of Bland–Altman analysis (mean bias and 95% limits of agreement of global FD: -0.005, -0.023 to 0.014, infarct FD: -0.011, -0.036 to 0.014, remote FD: -0.003, -0.029 to 0.023) are shown in Supplementary Fig. 1.

### Correlations between FD measures and ventricular parameters in each phase

Correlation analysis (Supplementary Table 1) revealed weak correlations between certain ventricular parameters and FD measures at each phase. In the acute phase, there was a weak positive correlation between left ventricular mass index (LVMi) and global and remote area FD measures (*r* = 0.28, *p* < 0.001). LVEDVi and edema size also showed weak positive correlations with global and remote area FD (LVEDVi with Global FD: *r* = 0.17, *p* = 0.02, with remote area FD: *r* = 0.18, *p* = 0.01; edema size with Global FD: *r* = 0.21, *p* = 0.003, with remote area FD: *r* = 0.18, *p* = 0.01). Infarct size was negatively correlated with infarct area FD value (*r* =  -0.15, *p* = 0.04). In the subacute and chronic phases, a weak positive correlation was consistently present between remote area FD and LVEDVi and LVSVi (subacute phase with LVEDVi: *r* = 0.16, *p* = 0.03, with LVSVi: *r* = 0.18, *p* = 0.01; chronic phase with LVEDVi: *r* = 0.15, *p* = 0.03, with LVSVi: *r* = 0.20, *p* = 0.01). In the chronic phase, a negative correlation was found between infarct area FD and infarct size (*r* =  -0.16, *p* = 0.02) and wall motion score (*r* =  -0.17, *p* = 0.02).

### FD as a marker of subsequent MACE

The univariable Cox regression analysis of subacute and chronic phase parameters with subsequent MACE is summarized in Table 3. Cox regression failed to identify any FD parameter at the acute phase that was significantly associated with MACE. However, it did identify the following subacute phase parameters: global FD, remote FD, Δglobal FD, and Δremote FD. Each of these factors was added one by one to a baseline model consisting of left ventricular ejection fraction, left ventricular end-diastolic volume index, infarct size, heart rate, NYHA class > 3, and post-surgical TIMI flow < 3 (Table 4). Only global FD and Δglobal FD significantly improved the C-statistic, IDI, and cNRI of the baseline model (Table 5), indicating that both were significantly associated with MACE (global FD, HR 0.89, 95% CI 0.82–0.97, *p* = 0.01; Δglobal FD, HR 0.91, 95% CI 0.84–0.98, *p* = 0.008).

**Table 3:** Subacute and chronic phase univariable Cox Regression Analysis of MACE

	Subacute phase		Chronic phase	
	Hazard Ratio (95% CI)	*p* value	Hazard Ratio (95% CI)	*p* value
Baseline characteristics				
Age	1 (0.97, 1.03)	0.82	1 (0.97, 1.03)	0.93
Male sex	0.77 (0.3, 1.99)	0.59	0.76 (0.3, 1.98)	0.58
Body mass index	0.9 (0.79, 1.03)	0.12	0.93 (0.82, 1.05)	0.24
Hypertension	1.27 (0.65, 2.5)	0.49	1.27 (0.65, 2.5)	0.49
Dyslipidemia	1.08 (0.47, 2.47)	0.87	1.08 (0.47, 2.48)	0.86
Diabetes mellitus	1.33 (0.68, 2.62)	0.41	1.33 (0.68, 2.62)	0.41
Anterior infarction	1.42 (0.62, 3.25)	0.40	1.41 (0.62, 3.23)	0.41
Pain-to-balloon time (h)	1 (1, 1.01)	0.11	1 (1, 1.01)	0.11
NYHA class > 3	1.57 (1.38, 4.46)	** < 0.001**	19.57 (4.38, 87.46)	** < 0.001**
Pre-pPCI TIMI flow < 3	1.81 (0.75, 4.37)	0.19	1.81 (0.75, 4.38)	0.19
Post-pPCI TIMI flow < 3	3.89 (1.61, 9.42)	**0.003**	4.15 (1.71, 10.1)	**0.002**
CMR parameters				
Heart rate	1.03 (1.01, 1.06)	**0.02**	1.02 (1, 1.05)	0.08
LVEF	0.96 (0.94, 0.99)	**0.005**	0.98 (0.97, 1)	**0.009**
LVMi	1.02 (1, 1.04)	**0.049**	1.03 (1, 1.05)	**0.03**
LVEDVi	1.03 (1.01, 1.04)	** < 0.001**	1.03 (1.02, 1.04)	** < 0.001**
LVESVi	1.03 (1.02, 1.04)	** < 0.001**	1.03 (1.02, 1.04)	** < 0.001**
LVSVi	1 (0.97, 1.04)	0.82	0.99 (0.96, 1.02)	0.611
Infarct size, per 10%	2.21 (1.65, 2.97)	** < 0.001**	2.14 (1.6, 2.87)	** < 0.001**
Infarct transmural extent, per 10%	1.19 (0.97, 1.45)	0.10	1.16 (0.96, 1.4)	0.13
LV thrombus	0.44 (0.06, 3.21)	0.42	0.82 (0.11, 5.97)	0.84
LV aneurysm	1.27 (0.58, 2.79)	0.55	1.38 (0.65, 2.94)	0.40
MVO/IMH	1.61 (0.84, 3.1)	0.15	0.93 (0.46, 1.88)	0.83
Papillary infarction	0.56 (0.20, 1.57)	0.27	0.56 (0.20, 1.58)	0.27
FD measures, per 1%				
Global FD	0.89 (0.82, 0.96)	**0.005**	0.93 (0.86, 1)	**0.04**
Infarct area FD	0.95 (0.9, 1)	0.07	0.95 (0.9, 1)	**0.04**
Remote area FD	0.95 (0.9, 1)	**0.04**	0.96 (0.91, 1.01)	0.13
ΔGlobal FD	0.85 (0.79, 0.91)	** < 0.001**	0.92 (0.86, 0.98)	**0.007**
ΔInfarct area FD	0.95 (0.88, 1.03)	0.23	0.96 (0.9, 1.02)	0.17
ΔRemote area FD	0.9 (0.84, 0.98)	**0.01**	0.96 (0.89, 1.02)	0.21

*TIMI* Thrombolysis in myocardial infarction, *LVEF* Left ventricular ejection fraction, *LVEDVi* Left ventricular end-diastolic volume index, *LVESVi* Left ventricular end-systolic volume index, *LVMi* Left ventricular mass index, *LVSVi* Left ventricular stroke volume index, *MVO* Microvascular obstruction, *IMH* Intramyocardial hemorrhage, *LVT* Left ventricular thrombus, *FD* Fractal dimension

**Table 4:** Subacute phase multivariable Cox Regression models of MACE

Models	Parameters	Hazard Ratio (95% CI)	*p* value
Model 1	LVEF	1.05 (1, 1.09)	**0.04**
	LVEDVi	1.02 (1, 1.03)	0.06
	Infarct size, per 10%	2.68 (1.75, 4.11)	** < 0.001**
	Heart rate	1.03 (1, 1.06)	0.07
	NYHA class > 3	4.62 (2.3, 8.17)	** < 0.001**
	Post-pPCI TIMI flow < 3	2.6 (0.99, 6.8)	0.051
Model 2	LVEF	1.05 (1.01, 1.1)	**0.02**
	LVEDVi	1.02 (1, 1.03)	**0.03**
	Infarct size, per 10%	2.67 (1.76, 4.04)	** < 0.001**
	Heart rate	1.03 (1, 1.06)	0.08
	NYHA class > 3	4.94 (2.87, 10.38)	** < 0.001**
	Post-pPCI TIMI flow < 3	2.72 (0.98, 7.56)	0.06
	Global FD, per 1%	0.89 (0.82, 0.97)	**0.01**
Model 3	LVEF	1.05 (1.01, 1.09)	**0.03**
	LVEDVi	1.02 (1, 1.04)	**0.03**
	Infarct size, per 10%	2.69 (1.76, 4.13)	** < 0.001**
	Heart rate	1.03 (1, 1.06)	0.09
	NYHA class > 3	3.71 (2.16, 7.92)	** < 0.001**
	Post-pPCI TIMI flow < 3	2.28 (0.83, 6.25)	0.11
	Remote area FD, per 1%	0.94 (0.88, 1)	0.07
Model 4	LVEF	1.06 (1.02, 1.11)	**0.009**
	LVEDVi	1.02 (1, 1.04)	**0.04**
	Infarct size, per 10%	2.42 (1.56, 3.74)	** < 0.001**
	Heart rate	1.03 (1, 1.06)	0.08
	NYHA class > 3	2.96 (1.49, 8.59)	** < 0.001**
	Post-pPCI TIMI flow < 3	3.41 (1.22, 9.51)	**0.02**
	ΔGlobal FD, per 1%	0.91 (0.84, 0.98)	0.008
Model 5	LVEF	1.05 (1.01, 1.1)	**0.03**
	LVEDVi	1.02 (1, 1.03)	0.06
	Infarct size, per 10%	2.62 (1.7, 4.03)	** < 0.001**
	Heart rate	1.03 (1, 1.06)	0.07
	NYHA class > 3	3.16 (2.11, 8.22)	** < 0.001**
	Post-pPCI TIMI flow < 3	2.74 (1.03, 7.3)	**0.04**
	ΔRemote area FD, per 1%	0.97 (0.89, 1.05)	0.42

*TIMI* Thrombolysis in myocardial infarction, *LVEF* Left ventricular ejection fraction, *LVEVi* Left ventricular end diastolic volume index, *FD* Fractal dimension

**Table 5:** C-statics, IDI and cNRI of multivariable Cox regression models with FD parameters

	C-statistics	*p* value	IDI (95% CI)	*p* value	cNRI (95% CI)	*p* value
Model 1	0.786 (0.708, 0.863)	/	/	/	/	/
Model 2	0.814 (0.751, 0.877)	**0.045**	0.142 (0.012, 0.208)	**0.03**	0.577 (0.385, 0.768)	**0.02**
Model 3	0.788 (0.712, 0.864)	0.40	0.008 (-0.195, 0.193)	0.90	0.127 (-1.187, 1.306)	0.99
Model 4	0.811 (0.738, 0.885)	**0.03**	0.077 (0.021, 0.133)	**0.02**	0.434 (0.112, 0.756)	**0.03**
Model 5	0.790 (0.714, 0.867)	0.08	0.003 (-0.033, 0.026)	0.99	0.263 (-0.602, 0.747)	0.78

*IDI* Integrated discrimination index, *cNRI* Continuous net reclassification improvement

**Fig. 5:**
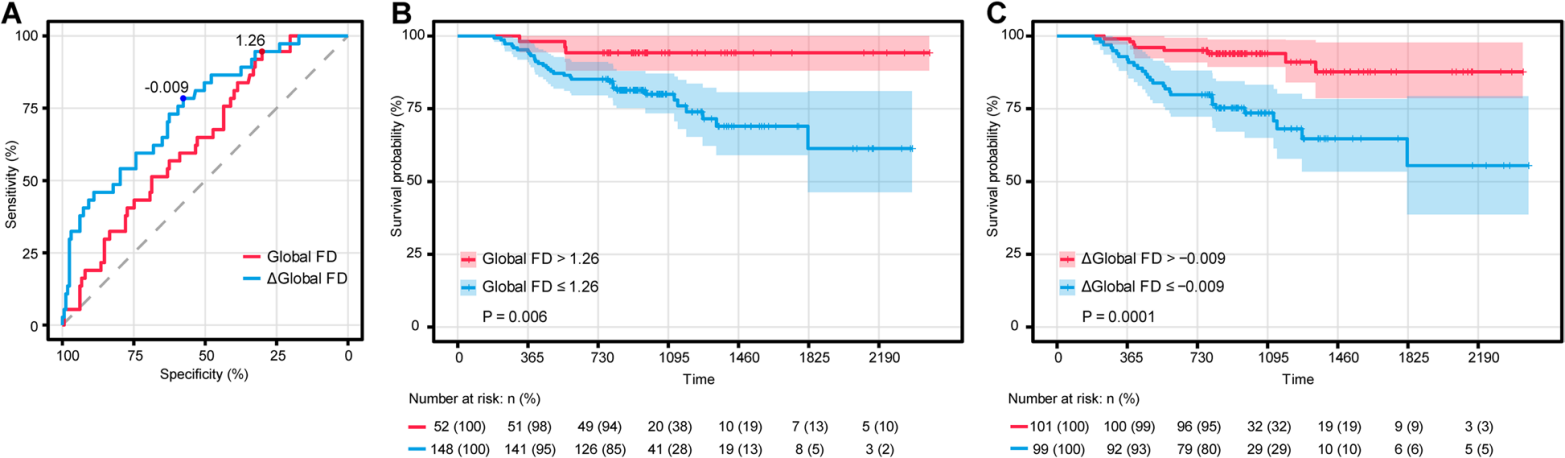
**A** Receiver operating characteristic analysis of global and Δglobal fractal dimension (FD) of the subacute phase for the prediction of major adverse cardiac events (MACE). The area under the curve (AUC) in the ROC analysis of global FD and Δglobal FD in the subacute phase for predicting MACE was 0.639 and 0.747, respectively. The cut-off values for global FD (1.26) and Δglobal FD (-0.009) were determined by the Youden index. **B** Kaplan–Meier curves for MACE based on the optimal cut-off of global FD in the subacute phase (> 1.26 versus ≤ 1.26), *p* = 0.006. **C** Kaplan–Meier curves for MACE based on the optimal cut-off of Δglobal FD in the subacute phase (> -0.009 versus ≤  -0.009), *p* = 0.0001

The area under the curve (AUC) in the ROC analysis of global FD and Δglobal FD in the subacute phase for predicting MACE was 0.639 and 0.747, respectively (Fig. 5A). At the optimal cut-off value of 1.26 for global FD, sensitivity was 94.6%, and specificity was 30.1%. At a cut-off of -0.009 for Δglobal FD, sensitivity was 78.4%, and specificity was 57.7%. Kaplan–Meier analysis showed that patients with parameters below these cut-off values were at a significantly greater risk of MACE during follow-up (Fig. 5B,C).

## Discussion

In this retrospective study, we assessed left ventricular trabecular complexity with fractal analysis in post-STEMI patients. Firstly, we observed a decrease in global and regional FD after STEMI, more pronounced in patients who developed MACE during the follow-up. Secondly, in multivariable Cox regression analysis, lower global FD and Δglobal FD represented independent risks for subsequent MACE. Global FD less than 1.26 at the subacute phase or Δglobal FD from the acute to subacute phase less than -0.009 would increase the risk of subsequent MACE.

Previous studies of fractal analysis were mainly conducted in patients with non-ischemic cardiomyopathy [4–6, 14–16]. In these studies, excessive trabeculation is associated with congenital heart disease or pressure overload in acquired cardiomyopathy. FD values were generally higher in patients than in healthy controls and correlated with impaired ventricular function and poor long-term outcomes.

In accordance with the spatial pattern observed in individuals afflicted with dilated cardiomyopathy (DCM) and the UK Biobank cohort [5], FD exhibited a comparable gradient that was lower at both the base and apex, reaching its zenith between the base and mid-ventricular levels in post-STEMI patients across all phases. Diverging from FD investigations pertaining to non-ischemic cardiomyopathy, this study analyzed regional FD values in the infarct and remote areas, eschewing the examination of basal and apical FD values. The variation in the measurement range is due to the fact that the scope of myocardial infarction depends on the cult vessels, rather than being confined solely to basal or apical levels of the heart. In all subjects post-STEMI, FD values exhibited a decline from the acute to chronic phases. The reduction in FD within the infarcted area was notably significant during the subacute and chronic phases. Given that myocardial infarctions typically implicate the endocardium [17, 18], the complexity of trabeculae in the infarcted region would diminish owing to the diminishing blood supply. The FD of the remote area also exhibited a reduction post-STEMI, particularly in subjects experiencing MACE, and this alteration was in harmony with the overall FD. This observation suggests that the temporal alteration in trabecular complexity in post-STEMI patients is intricate. The diminished shear strain [19], compromised coronary vasodilator function, and compromised oxygenation [20, 21] in both the infarct and remote areas, may underlie such morphological changes.

Infarct size has been established as a robust determinant of outcome [22]; however, in this study, FD was also associated with MACE during long-term follow-up, signifying the complexity of trabeculation. In the acute phase post-STEMI, global FD (1.253 ± 0.049) exceeded the previously reported global FD value in Chinese normal controls (1.192 ± 0.032, 1.195 ± 0.030, 1.205 ± 0.031) [4, 6, 23]. This observation aligns with a study on left ventricular trabeculation [24], indicating LV hypertrabeculation associated with MACE in the general population. However, post-STEMI, the correlation between FD and MACE reversed. Patients with a lower global FD value in the subacute phase, especially < 1.26, exhibited a heightened risk of subsequent MACE. The cause of more pronounced trabeculae loss in patients experiencing subsequent MACE within the first month after acute STEMI requires further investigation. The physiological role of trabeculae in post-STEMI patients remains unclear. The Δglobal FD from acute to subacute phases, showing -0.009 (a decrease exceeding 0.009), increased the risk of subsequent MACE significantly (*p* = 0.0001). A greater reduction in global FD from the acute to subacute phase corresponded to a higher long-term risk of MACE.

In this study, global FD outperformed regional FD in the analysis of subsequent MACE. The cut-off value for global FD was also assessed. A global FD value < 1.26 exhibited high sensitivity (94.6%) in predicting subsequent MACE. Given its high reproducibility and ease of measurement, global FD holds promise for clinical application. The cut-off for global FD in subacute phase risk assessment warrants further prospective evaluation.

This study has several limitations, including as follows: Firstly, due to its retrospective nature, only patients who underwent pPCI for STEMI and possessed CMR data in the acute, subacute, and chronic phases were eligible for inclusion. This may introduce a potential selection bias. Secondly, it is important to note that FD can be influenced by hemodynamic factors and valvular anatomy, aspects that were not considered in the scope of this investigation. Thirdly, the participants in this study were predominantly male, with 91% representation. This gender imbalance may introduce bias and subsequently limit expressiveness for female patients.

In conclusion, the application of fractal dimension through CMR offers a quantitative assessment of trabeculae complexity, temporally linked to the clinical outcomes of acute STEMI patients. Notably, global FD at the subacute phase post-STEMI independently demonstrated an association with subsequent MACE.

### Supplementary Information

**Supplementary Material 1.**

## Data Availability

The datasets used and/or analyzed during the current study are available from the corresponding author on reasonable request.

## Abbreviations

CMR: Cardiac magnetic resonance
FD: Fractal dimension
IMH: Intramyocardial hemorrhage
MACE: Major adverse cardiovascular event
MVO: Microvascular obstruction
pPCI: Primary percutaneous coronary intervention
STEMI: ST elevation myocardial infarction
TIMI: Thrombolysis in myocardial infarction
