# Supplementary material for: Fractal analysis of left ventricular trabeculae in post-STEMI: from acute to chronic phase
Source: Insights Imaging. 2024 Mar 18;15:75. doi: 10.1186/s13244-024-01641-8 (PMC10948656; doi:10.1186/s13244-024-01641-8)
Supplement: Supplementary file 1 — Supplementary Material 1. [file 13244_2024_1641_MOESM1_ESM.pdf]

Fractal Analysis of Left Ventricular Trabeculae in post-STEMI: from Acute to Chronic Phase

ELECTRONIC SUPPLEMENTARY MATERIAL

CMR Acquisition Protocol

Images were acquired with following 3.0 Tesla MRI scanners: Ingenia, Philips Healthcare, Best, The Netherlands; Ingenia CX, Philips Healthcare, Best, The Netherlands; Achieva, Philips Healthcare, Best, The Netherlands, MAGNETOM Verio, Siemens Healthcare, Germany; Discovery MR750W, GE Health Medical, United States. The routine analysis included steady-state free-precession (SSFP) cine images (short-axis stack from left ventricle base to apex, two-chamber, three-chamber, and four-chamber planes), T2-weighted imaging with fat suppression (T2WFS) images (short-axis stack from base to apex), and late gadolinium enhancement (LGE) images (short-axis stack matched with T2WFS) 10 minutes after gadolinium-DTPA (Magnevist Bayer Healthcare, Berlin, Germany) injection. The sequences of the technique performed as follows:

Philips Ingenia, Ingenia CX, and Achieva: (1) b-SSFP cine sequences: repetition time (TR) = 2.8 ms, echo time (TE) = 1.4 ms, slice thickness = 7 mm, acquired matrix = 1.2 mm x 1.2 mm,

phases per cardiac cycle = 30, field of view (FOV) = 300 mm x 300 mm; (2) T2WFS sequence: TR = 1714 ms, TE = 75 ms, inversion time (TI) = 220ms, slice thickness = 10 mm, FOV = 300 mm x 300 mm, acquired matrix = 0.89 mm x 0.89 mm; and (3) PSIR sequence: TR = 6.1 ms, TE = 3 ms, slice thickness = 10 mm, FOV = 300 mm x 300 mm, acquired matrix = 1.6 mm x 1.9 mm.

Siemens Verio: (1) b-SSFP cine sequences: TR = 42.0 ms, TE = 1.25 ms, slice thickness = 8 mm, acquired matrix = 1.25 mm x 1.25 mm, phases per cardiac cycle = 30, field of view (FOV) = 240 mm x 240 mm; (2) T2WFS sequence: TR = 722 ms, TE = 30 ms, TI = 220ms, slice thickness = 8 mm, FOV = 260 mm x 260 mm, acquired matrix = 1.01 mm x 1.01 mm; and (3) PSIR sequence: TR = 660 ms, TE = 3.7 ms, slice thickness = 8 mm, FOV = 250 mm x 250 mm, acquired matrix = 0.98 mm x 0.98 mm.

GE MR750W: (1) b-SSFP cine sequences: TR = 3.47 ms, TE = 1.51 ms, slice thickness = 10 mm, acquired matrix = 1.09 mm x 1.09 mm, phases per cardiac cycle = 30, field of view (FOV) = 280 mm x 280 mm; (2) T2WFS sequence: TR = 2R-R, TE = 72 ms, TI = 220 ms, slice thickness = 10 mm, FOV = 280 mm x 280 mm, acquired matrix = 1.09 mm x 1.09 mm; and (3) PSIR sequence: TR = 6.62 ms, TE = 3.10 ms, slice thickness = 10 mm, FOV = 260 mm x 260 mm, acquired matrix = 1.01 mm x 1.01 mm.

CMR Image assessment

The volume, mass, and function of the left ventricle were determined using short-axis cine images. Infarct size, edema, microvascular obstruction (MVO), and intramyocardial hemorrhage（IMH) were analyzed by T2WFS and LGE images. Edema area was identified with the mean + 2 standard deviation method, and the infarct area was identified with the full width at half-maximum (FWHM) method. Infarct transmural extent was calculated from LGE images as a percentage by dividing infarct wall thickness by regional wall thickness.

Supplementary Table 1 Correlation between fractal dimension (FD) with left ventricular characteristics

	Global FD		Infarct area FD		Remote area FD	
	r	*p*-value	r	*p*-value	r	*p*-value
Acute phase						
Heart rate	-0.02	0.81	-0.10	0.16	-0.09	0.22
LVEF	-0.07	0.36	0.09	0.23	-0.03	0.68
LVEDVi	0.17	**0.02**	0.01	0.89	0.18	**0.01**
LVESVi	0.15	**0.03**	-0.05	0.46	0.12	0.08
LVSVi	0.08	0.28	0.08	0.24	0.13	0.08
LVMi	0.28	**<0.001**	0.11	0.11	0.27	**<0.001**
Infarct size	0.12	0.10	-0.15	**0.04**	0.07	0.36
Edema size	0.21	**0.003**	-0.08	0.24	0.18	**0.01**
Infarct transmural extent	0.14	0.06	-0.12	0.10	0.18	0.07
Subacute phase						
Heart rate	-0.02	0.75	-0.04	0.62	-0.04	0.59
LVEF	-0.03	0.67	0.08	0.24	0.02	0.79
LVEDVi	0.09	0.21	-0.06	0.40	0.16	**0.03**
LVESVi	0.07	0.29	-0.09	0.21	0.06	0.37
LVSVi	0.11	0.12	0.02	0.77	0.18	**0.01**
LVMi	0.03	0.70	0.10	0.18	0.03	0.67
Infarct size	-0.01	0.92	-0.08	0.28	-0.05	0.45
Infarct transmural extent	0.11	0.13	-0.09	0.18	0.11	0.13
Chronic phase						
Heart rate	0.04	0.55	-0.04	0.59	-0.01	0.89
LVEF	0.05	0.52	0.10	0.14	0.04	0.59
LVEDVi	0.09	0.23	-0.07	0.29	0.15	**0.03**
LVESVi	0.02	0.75	-0.10	0.15	0.07	0.35
LVSVi	0.14	0.05	0.02	0.75	0.20	**0.01**
LVMi	0.02	0.73	0.05	0.51	0.08	0.25
Infarct size	-0.04	0.53	-0.16	**0.02**	0.03	0.70
Infarct transmural extent	0.07	0.34	-0.13	0.07	0.10	0.14
Wall motion score	0.01	0.85	-0.17	**0.02**	0.04	0.53

Note: LVEF = left ventricular ejection fraction, LVEDVi = left ventricular end-diastolic volume index, LVESVi = left ventricular end-systolic volume index, LVSVi = left ventricular stroke volume index, LVMi = left ventricular mass index, FD = fractal dimension.

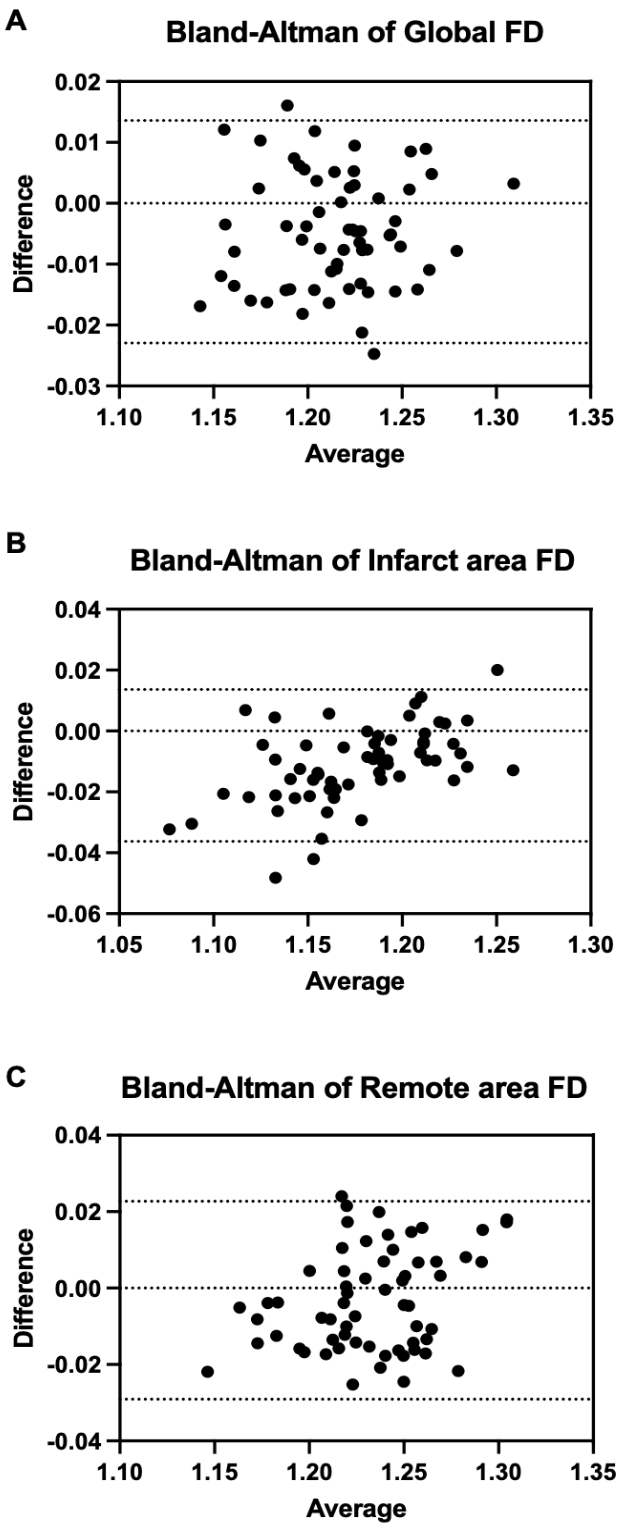

Supplementary Figure 1. **A-C**: Bland-Altman analysis of global, infarct area and remote area fractal dimension.

Note: FD = Fractal Dimension
